# Inactivation of the Lateral Hypothalamus Attenuates Methamphetamine-Induced Conditioned Place Preference through Regulation of Kcnq3 Expression

**DOI:** 10.3390/ijms23137305

**Published:** 2022-06-30

**Authors:** Min Liu, Xu Tan, E Liu, Zhaofang Hang, Ruiheng Song, Shouhong Mu, Weikai Han, Qingwei Yue, Jinhao Sun

**Affiliations:** Department of Anatomy, School of Basic Medical Sciences, Shandong University, Jinan 250012, China; liuminsdu@mail.sdu.edu.cn (M.L.); 201720430@mail.sdu.edu.cn (X.T.); liue@bjmu.edu.cn (E.L.); hangzhaofang@mail.sdu.edu.cn (Z.H.); songruiheng@mail.sdu.edu.cn (R.S.); mushouhong@mail.sdu.edu.cn (S.M.); hanweikai@mail.sdu.edu.cn (W.H.)

**Keywords:** methamphetamine, lateral hypothalamus, addiction, synaptic plasticity, Kcnq3 channel

## Abstract

Repeated administration of methylamphetamine (MA) induces MA addiction, which is featured by awfully unpleasant physical and emotional experiences after drug use is terminated. Neurophysiological studies show that the lateral hypothalamus (LH) is involved in reward development and addictive behaviors. Here, we show that repeated administration of MA activates the expression of c-Fos in LH neurons responding to conditioned place preference (CPP). Chemogenetic inhibition of the LH can disrupt the addiction behavior, demonstrating that the LH plays an important role in MA-induced reward processing. Critically, MA remodels the neurons of LH synaptic plasticity, increases intracellular calcium level, and enhances spontaneous current and evoked potentials of neurons compared to the saline group. Furthermore, overexpression of the potassium voltage-gated channel subfamily Q member 3 (Kcnq3) expression can reverse the CPP score and alleviate the occurrence of addictive behaviors. Together, these results unravel a new neurobiological mechanism underlying the MA-induced addiction in the lateral hypothalamus, which could pave the way toward new and effective interventions for this addiction disease.

## 1. Introduction

Drug addiction is a chronic disease, which is accompanied by high relapse rates, compulsive drug-seeking, and forceful withdrawal reactions [1]. Addictive drugs, including methamphetamine (MA), as one of the addictive drugs of abuse, have been a global health threat for fairly long time [2]. The location of the lateral hypothalamus (LH) is in the midbrain region partly and plays a crucial part in maintaining physiological and behavioral homeostasis, which has been involved in reward course and addictive behaviors, particularly in regulating diet [3]. Recently, a growing number of studies are reporting that the abuse of addiction drugs, such as ethanol and cocaine can activate LH neurons [4,5] and produce different regulation effects [6,7,8]. Importantly, it has been observed that MA can activate neurons in the LH region with chronic MA self-administration [9]. However, the further specific mechanism through which MA affects LH regulation is still elusive.

Previous studies have clearly demonstrated that LH neurons regulate reward and motivation, and an increasing number of studies have also emphasized the significance of the LH. In addition, a recent study demonstrated a GABAergic population in the LH, which regulates reward-seeking or feeding [10]. Importantly, manipulating the activation of LH neurons can regulate extinguished morphine-seeking behavior [11], as examined by the expression of c-Fos, which is an immediate early gene [12,13]. In turn, as with the activation of neurons, the intrinsic excitability and intracellular Ca^2+^ are also enhanced [14] in cocaine self-administering animals [15]. Interestingly, it has been indicated that the neuronal excitability is controlled by Kcnq channels, members of the K^+^ channel family, which play an important role in the hyperpolarization and reduction in synaptic firing [16,17]. In addition, multiple studies have implicated potassium voltage-gated channel subfamily Q member 3 (Kcnq3) channel dysfunction in multiple monogenic neurodevelopmental disorders [18,19], such as benign familial neonatal epilepsy (BFNE) and benign familial infantile epilepsy (BFIE) [20], and so Kcnq3-related developmental disability remains to be defined. Additionally, recent studies reveal that chronic drinking can affect the Kcnq channels in the nucleus accumbens and the Kcnq channels are targets for decreasing alcohol consumption [21], which also means that these K+ channels may regulate the risk of developing an alcohol use disorder [22,23]. Thus, we hypothesize that targeting K+ channels may be efficacious in the treatment of other addictions, including MA addiction. Furthermore, drug-induced structural plasticity of dendritic spines in the brain’s reward circuit is critical for aberrant drug-related learning and addictive behaviors [24]. For example, MA-induced augmented spine density is associated with drug-related memory acquisition [25]. Moreover, the inhibition of the formation of dendritic spines can disrupt established MA-associated memories and addictive behaviors [26]. However, the effects of MA on LH neuron dendritic spines remain to be elucidated.

Based on these considerations, the objective of this study was to decipher the effect of the LH on MA addiction behavior and its underlying mechanism. In our study, we first determined the expression of c-Fos, a marker of neuronal excitation in the LH. We also determined Ca^2+^ levels in the LH by means of the Gcamp6 AAV virus, as well as the levels of the Ca^2+^-related protein p-CREB. Furthermore, we proved the function of the LH by chemogenetic inhibition and detected the synaptic plasticity of the LH in addicted mice. In addition, we tested whether MA can change neuronal excitability using a patch clamp. Finally, the Kcnq3 overexpression AAV virus was used to confirm that Kcnq3 regulated the occurrence of addictive behaviors. Overall, our study indicated that Kcnq3 in the LH is conducive to the occurrence of MA-induced addiction behavior, which could be a potential target for the treatment of MA addiction.

## 2. Results

### 2.1. LH Neurons Were Activated in MA-Induced CPP Mice

**Figure 1:**
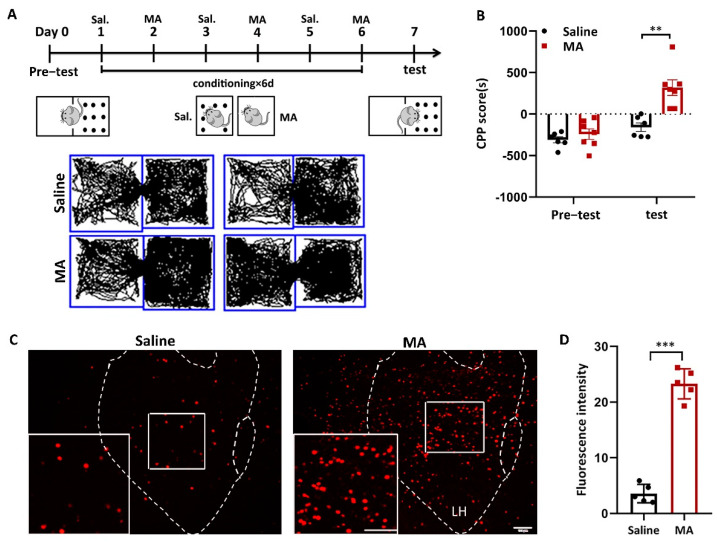
MA-activated LH neurons in MA-induced CPP mice. (**A**) Experimental timeline. (**B**) Average CPP scores. (Saline, *n* = 6. MA, *n* = 7) (**C**) c-Fos^+^ neurons were in the LH. Scale bars, 100 μm. (**D**) Quantification of c-Fos^+^ cells in the LH (*n* = 6). Data represent the means ± SEM. ** *p* < 0.01, *** *p* < 0.001 vs. saline by *t* test.

We used a conditioned place preference (CPP) test to evaluate MA-induced addiction. The animal behavior video analysis system was used to record the time the mice stayed in different boxes to evaluate the success of MA-induced CPP. Subsequently, 2 mg/kg MA was injected (i.p.) into mice according to the training paradigm (Figure 1A). CPP score (calculated as the time in the white box minus the time in the black box) was used to assess addictive result directly, and the results revealed that the CPP score of the MA group was remarkably higher (Figure 1B, *p* < 0.01) after the paradigm, demonstrating that the MA group displayed more preference to bright boxes than the saline group. To research the function of LH neurons in MA-induced CPP behavior, we detected the impact of MA on the expression of c-Fos, a marker of neuronal activation [27]. The results showed that the expression of c-Fos in the LH was significantly increased in the MA group (*p* < 0.001) after exposure to the conditioned context (Figure 1C,D). These results revealed that MA exposure greatly increased the activity of neurons in the LH during CPP.

### 2.2. MA Increased p-CREB and Intracellular Ca^2+^ Levels in LH

**Figure 2:**
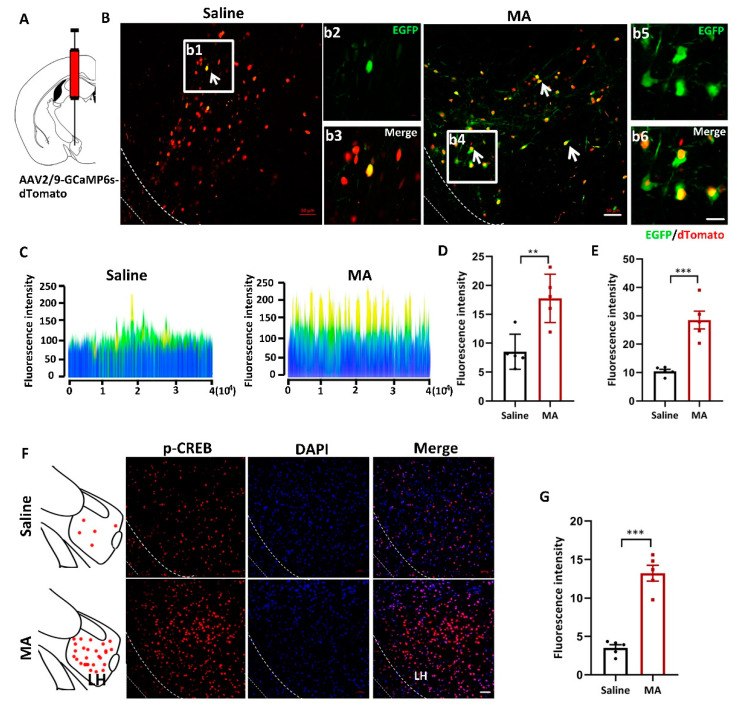
MA increased the intracellular Ca^2+^ levels in MA-induced CPP mice. (**A**) Injection diagram of the virus into the bilateral LH. (**B**) Ca^2+^ levels indicated by AAV-dTomato-Gcamp6-EGFP virus in the saline and MA groups. b2 and b3 (scale bars, 20 μm) are the amplification of selected areas from b1 (scale bars, 50 μm). b5 and b6 (scale bars, 20 μm) are the amplification of selected areas from b4 (scale bars, 50 μm). (**C**) 2.5 D rebuilt the picture through ZEN blue software. (**D**,**E**) Quantification of the fluorescence intensity of EGFP^+^ cells in the LH (J: original, K: magnified pictures, respectively, *n* = 5). (**F**,**G**) Immunofluorescence and quantification of p-CREB expression in the different groups (*n* = 5 per group); scale bars, 50 μm. Data represent the means ± SEM. ** *p* < 0.01, *** *p* < 0.001 vs. saline by *t* test.

To visualize the changes in intracellular Ca^2+^ levels, mice received injections of the AAV2/9-CMV-GCaMP6s-P2A-nls-dTomato virus 300 nL into the LH (Figure 2A). LH neurons infected with the AAV-GCaMP6s virus were labeled and visualized by tdTomato in the saline and MA groups. EGFP (green) indicated the level of intracellular Ca^2+^ (Figure 2B; b2 and b3 are magnified areas from b1, b5 and b6 are magnified areas from b4). The fluorescence was revealed by a 2.5D picture rebuilt by ZEN blue software (Figure 2C). The fluorescence intensity of EGFP was significantly increased in mice treated with MA compared with the saline group (Figure 2D,E, representing the original pictures, *p* < 0.01 and magnified pictures, *p* < 0.001, respectively). To detect the effect of MA on the CREB-Ca^2+^ signaling pathway, we detected the expression of p-CREB, which is related to intracellular Ca^2+^ levels, and we found that MA upregulated the expression of p-CREB levels compared with saline (Figure 2F,G, *p* < 0.001).

### 2.3. Inhibition of LH Reversed MA-Induced CPP in Mice

**Figure 3:**
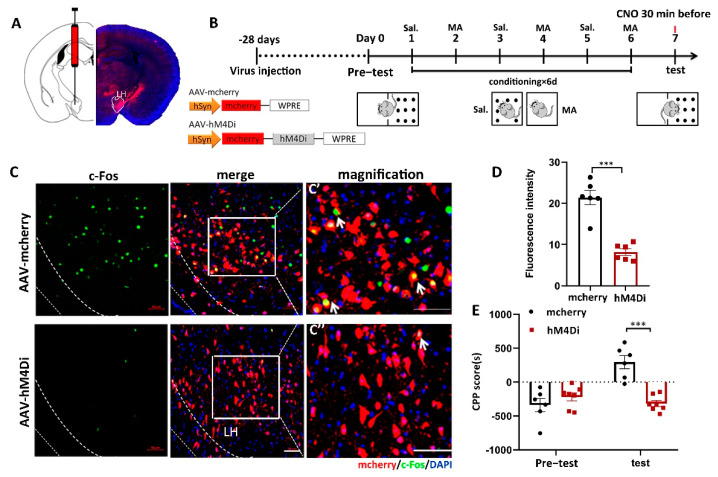
The influence of the inhibition of LH neurons on the CPP scores in MA-induced mice. (**A**) Diagram of the injection of virus into the bilateral LH. (**B**) Experimental timeline. (**C**) The expression of mCherry (red) or hM4Di-mCherry (red) was in the LH, and the mCherry co-labeled neurons of c-Fos (green) were in the LH. Scale bars, 50 μm. (**C′**,**C′′**) Magnified pictures. Scale bars, 50 μm. (**D**) Quantification of c-Fos expression in the mCherry and hM4Di groups (*n* = 6 per group). (**E**) Average CPP scores. (Saline, *n* = 6. MA, *n* = 7) Data are reported as the mean ± SEM. *** *p* < 0.001 vs. saline group by *t* test.

To address the function of LH neurons in MA-induced CPP, we confirmed the effect of chemogenetic (designer receptors exclusively activated by designer drugs (DREADD)) inactivation of LH neurons on MA-induced CPP in mice. In the present study, we injected two AAVs, AAV9-hSyn-hM4D (human M4 muscarinic receptor) (Gi)-mCherry or AAV9-hSyn-mCherry, into the LH bilaterally (Figure 3A). Thus, hM4Di receptor expression only occurred in LH neurons, which could bind to clozapine-N-oxide (CNO) and inhibit the activity of neurons. The two groups were injected with CNO 30 min before the test. The two groups were subjected to behavioral paradigms, as shown in Figure 3B. Consequently, in the case of giving MA at the same time, the expression of c-Fos was significantly inhibited by AAV-hM4Di compared to AAV-mCherry, as we expected (Figure 3C,D, *p* < 0.001). Meanwhile, at the behavioral level, the decreased excitation in LH neurons prevented MA-induced CPP (Figure 3E, *p* < 0.001). In terms of CPP scores, changes in CPP scores were almost completely suppressed by AAV-hM4Di. This proof indicated that the excitation of LH neurons was essential for the conditioned place preference in MA mice.

### 2.4. Neuronal Excitatory Transmission Was Increased in MA-Treated Mice

**Figure 4:**
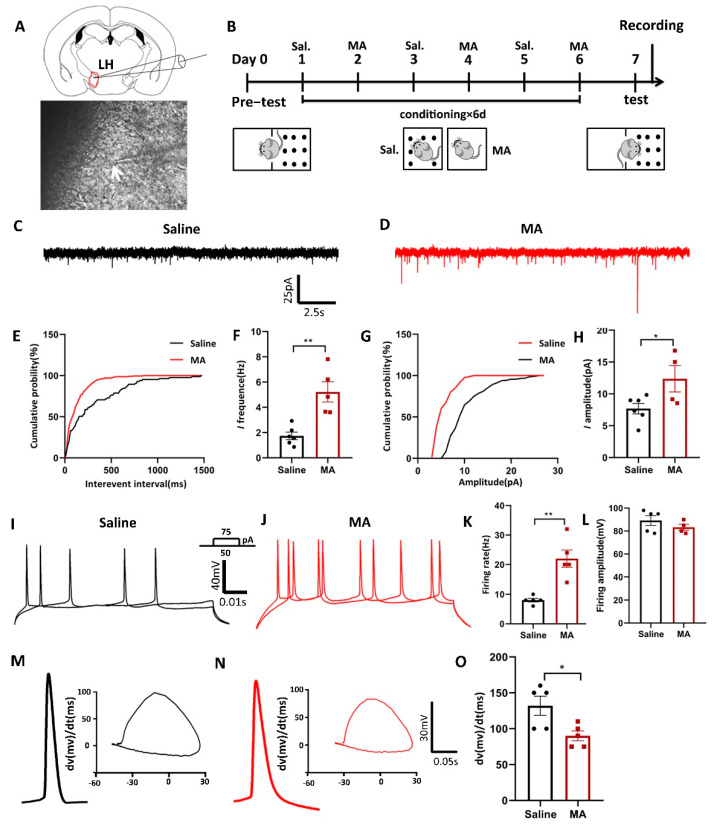
Neuronal excitatory transmission was increased in MA-exposed mice. (**A**) Patch clamp experiment of LH. (**B**) Experimental timeline. (**C**–**H**) The frequency and amplitude of spontaneous current in the saline and MA groups. (Frequency: *n* = 6 in the saline group, *n* = 5 in the MA group. Amplitude: *n* = 6 in the saline group, *n* = 4 in the MA group.) (**I**–**L**) The frequency and amplitude of evoked potentials in the saline and MA groups. (Frequency: *n* = 5 in the saline group, *n* = 5 in the MA group. Amplitude: *n* = 5 in the saline group, *n* = 4 in the MA group.) (**M**–**O**) Phase position analysis of the saline and MA groups (*n* = 5). Data are reported as the mean ± SEM. * *p* < 0.05 and ** *p* < 0.01 vs. saline group by *t* test.

Furthermore, given the notion that increased neuronal activity might regulate the progress of addiction [28], we conducted ex vivo electrophysiological research (Figure 4A) to reveal the changes in current and evoked potentials in MA-induced addiction mice in the LH after the paradigm of Figure 4B. We found that MA increased both the spontaneous current frequency (Figure 4C,F) and the amplitude (Figure 4G,H) compared with the saline group (frequency: *p* < 0.01; amplitude: *p* < 0.05). Moreover, for the evoked firing potential, which is the electrophysiological response of LH neurons to current steps by 50 and 75 pA, MA increased firing frequency (*p* < 0.01) but did not affect the amplitude (*p* > 0.05) compared with the saline group (Figure 4I–L). In addition, in the process of evoked action potential release, we could analyze the changes in the first derivative and phase, which could be obtained at a specific time or at a specific membrane. This analysis could be performed at the millisecond level, allowing us to accurately understand the evoked action potential dynamics. In the present study, we found that the dv(mv)/dt(ms) was 90 ± 6.892 in the MA group compared with 132 ± 13.19 in the saline group (Figure 4M–O, *p* < 0.05) in the process of depolarization, which meant that MA could lead to a reduction in cell membrane permeability. These results suggested that MA could enhance neuronal excitatory transmission in the LH neuron microcircuit, which might then contribute to the MA-induced CPP behavior observed in mice.

### 2.5. MA-Induced Structural Plasticity in LH

**Figure 5:**
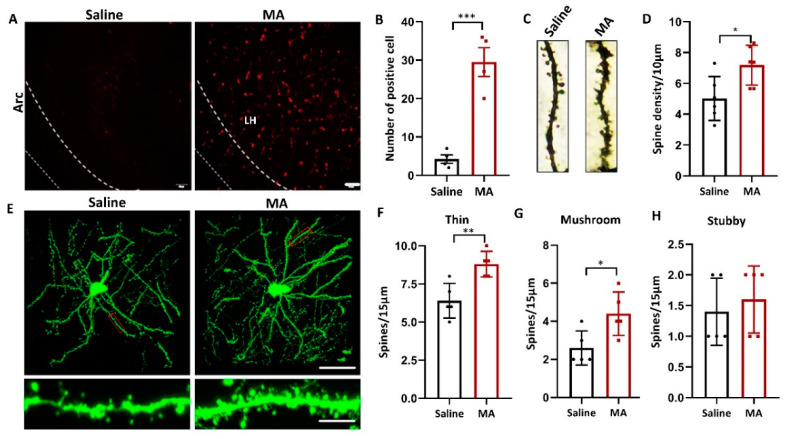
MA regulated LH neuron structural remodeling. (**A**,**B**) Immunofluorescence and quantification of Arc expression in the different groups (*n* = 5 per group). (**C**,**D**) Representative images and total dendritic spines in LH neurons detected by Golgi staining (*n* = 6 neurons/group, sampled from 3 mice). (**E**) Top: Representative morphology of LH neurons in each group. (*n* = 5 neurons/group, sampled from 3 mice.) Bottom: Enlargement of the red rectangle of the top. The images were obtained by confocal Z-stack scanning. Scale bar = 40 μm. Scale bar = 5 μm. (**F**–**H**) Densities of thin, mushroom, and stubby spines (*n* = 5 neuron dendrites/group, sampled from 3 mice/group). Data represent the mean ± SEM. * *p* < 0.05, ** *p* < 0.01, *** *p* < 0.001 vs. saline by *t* test.

Since structural plasticity was associated with MA- and cocaine-induced CPP [26,29], we wondered whether MA also influenced the structural plasticity of LH neurons. To research whether MA causes structural plasticity in LH neurons, we investigated the influence of MA on the level of recombinant activity-regulated cytoskeleton-associated protein (Arc), a marker of neuroplasticity [30]. Our results revealed that compared with that in the saline group, the expression of Arc was increased in the MA group (Figure 5A,B, *p* < 0.001). The total spine density of the LH was greatly increased in the MA group compared to the saline group (Figure 5C,D, *p* < 0.05) as detected by Golgi staining. To refine the structure of dendritic spines, a viral admixture was used to label LH neurons (Figure 5E). The results demonstrated that MA induced an increase in the densities of thin (Figure 5F, *p* < 0.01) and mushroom spines (Figure 5G, *p* < 0.05); nevertheless, the stubby spine density was unaffected (Figure 5H, *p* > 0.05). These results indicated that MA could regulate LH neuron structural remodeling, which was associated with MA-induced addiction behavior.

### 2.6. Overexpression of Kcnq3 Could Prevent MA-Induced Addictive Behavior

**Figure 6:**
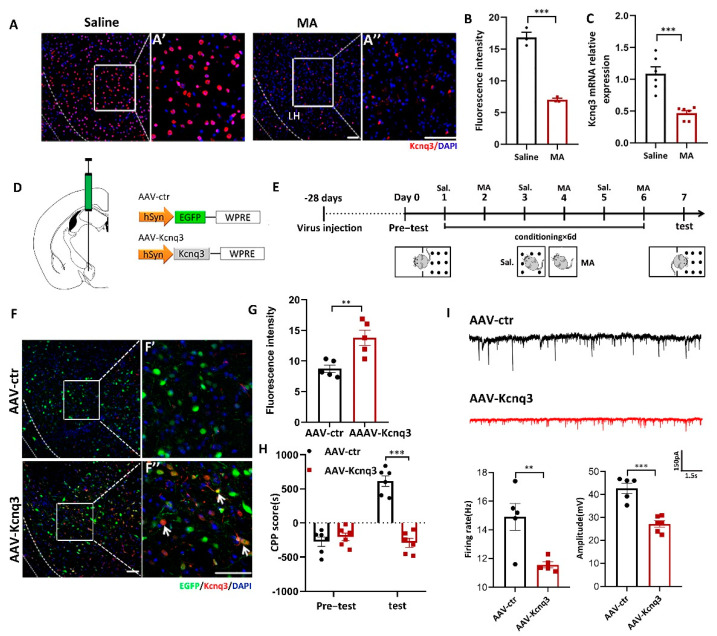
Overexpression of Kcnq3 prevented MA-induced CPP. (**A**,**B**) Immunofluorescence and quantification of Kcnq3 expression in the saline and MA groups (*n* = 3 per group). Scale bar = 50 μm. (**C**) The mRNA level of Kcnq3 detected by qRT–PCR (*n* = 6 per group). (**D**) Construct of AAV-virus. (**E**) Experimental timeline. (**F**) The injection site and the expression of EGFP (green) or Kcnq3 (red) in the LH; scale bars, 50 μm. (**F′**,**F′′**) Magnified pictures, scale bars, 50 μm. (**G**) Quantification of Kcnq3 expression in the AAV-ctr and AAV-Kcnq3 groups (*n* = 5 per group). (**H**) Average CPP scores (saline, *n* = 6. MA, *n* = 6). (**I**) The frequency and amplitude of EPSCs in labeling neurons of the LH. Data are reported as the mean ± SEM. ** *p* < 0.01, *** *p* < 0.001 vs. saline or AAV-ctr by *t* test.

To determine whether MA could affect the expression of Kcnq3, immunofluorescence and real-time fluorescence quantitative experiments were conducted. The results demonstrated that MA decreased the expression of Kcnq3 at the protein (Figure 6A,B, *p* < 0.001) and mRNA levels (Figure 6C, *p* < 0.001). We then constructed overexpression viruses for AAV-Control and AAV-Kcnq3 (Figure 6D). The saline and MA groups experienced the behavioral paradigm displayed in Figure 6E. The results indicated that Kcnq3 was overexpressed in the LH in the AAV-Kcnq3 group but not in the AAV-Control group (Figure 6F,G, *p* < 0.01 F′ and F′′ are magnified areas from F). Meanwhile, at the behavioral level, overexpressed Kcnq3 in the LH prevented MA-induced CPP (Figure 6H, *p* < 0.001). In a next step, we quantified the frequency and amplitude of excitatory postsynaptic currents (EPSCs) in labeling neurons of the LH by performing whole-cell recordings in brain slices. We found that EPSCs’ frequency amplitudes were decreased in AAV-Kcnq3 infected neurons compared to AAV-ctr neurons (Figure 6I, *p* < 0.05 and *p* < 0.01). Together, these results suggested that MA could reduce the expression of the Kcnq3 protein, which may be closely related to CPP caused by MA. Thus, improving the level state of the Kcnq3 channel could reinstate MA-induced addiction.

## 3. Discussion

In the present study, we examined the roles of the LH in MA-associated addiction behavior and related mechanisms. Our results demonstrated that the LH was a critical nucleus in the neural networks that orchestrated the addiction formation. Here, we identified that repeated administration of MA activated the expression of c-Fos in LH neurons in response to conditioned place preference (CPP). With chemogenetics, we demonstrated that inhibition of LH could disrupt the addiction behavior. Critically, MA remodels LH synaptic plasticity, increases intracellular calcium levels, and enhances electrical signals in neurons. Finally, overexpressed Kcnq3 could reverse the CPP score and alleviate the occurrence of addictive behavior. Taken together, our data provide critical support for the viewpoint that the LH plays a key role in the progression of MA addiction, which could pave the way toward new and efficient interventions for this addiction disorder.

Overall, our data suggest that MA could activate LH neurons and increase intracellular calcium levels in LH neurons. The results were in keeping with previous reports that c-Fos gene excitation was considered a neuronal marker of strong firing activity [31] and was activated by addictive drugs [32]. In addition, c-Fos expression requires a powerful increase in calcium influx [33] and is related to downstream signal changes that regulate gene expression [34]. In addition, given that p-CREB activity in the related region facilitates motivation for cocaine [15] and enhanced excitability of related neurons [14], we initially hypothesized that p-CREB activity in the LH would enhance the motivation for MA. Indeed, we found that MA could drive the signaling pathway of Ca^2+^-CREB, thus promoting neuron excitability in MA-addicted mice [35]. Interestingly, the LH has been widely researched for its concerns in reward development and feeding behaviors [36,37,38]; in particular, it has been proposed that manipulating LH neurons might accelerate LH- and VTA-induced self-stimulation [39]. The LH has been suggested to be responsible for sustaining positive reinforcement on MA addiction in our study. Indeed, neuronal activity could be inhibited by chemogenetics, and the expression of c-Fos was inhibited in the hM4Di group; meanwhile, the inhibition of the LH could disrupt the addiction behavior. All of the above studies supported our finding that the LH was involved in mediating MA-induced CPP.

In particular, after the MA CPP paradigm, we could see more complex dendrites, the dendrite length and branches were increased, and the same with the density of dendrite spines, particularly the thin and mushroom spines in the LH. Our results were in accordance with previous results that MA exposure increased the density of thin and mushroom spines in the dorsal striatum [40]. It was also reported that the dendritic structures of neurons were intricate dendritic branches and plentiful dendritic spines; these branches and spines were susceptible to morphological changes after drug exposure [6,7,8]. Additionally, the increase in spine density has been hypothesized to be related to the formation of drug-associated memory [25], further supporting our hypothesis that CPP induced by MA might be related to the structural plasticity of LH neurons. These structural changes in dendrites in the LH were also researched from a functional viewpoint level through patch-clamp experiments, and consistently, we found that MA increased the spontaneous current amplitude, frequency, and evoked firing rate. Together, these findings suggested that MA-induced CPP was related to an alteration in the excitatory transmission [41], in keeping with the idea that drug-evoked synaptic plasticity could strengthen excitatory afferents onto the downstream neurons exposed to an addictive drug [42], which was associated with the enhancement of electrical transmission [43].

It is important to note that cocaine abuse is known to increase the level of intracellular calcium, which reportedly could cause a widely described suppression of Kcnq7 ion channel activity, leading to hyperexcitability [44]. Considering our above study that MA leads to the excitability of neurons in the LH, we wondered whether MA also improved the excitability of neurons by inhibiting the expression of Kcnq3. Correspondingly, the mechanism underlying the addiction increase in neural excitability might be due to changes in ionic channels that were the basis of the changes in AP firing [45]. Indeed, MA downregulated the Kcnq3 level, and interestingly, in this situation, we overexpressed Kcnq3 by the AAV virus, which could lead to great changes in the CPP score and EPSCs. This finding is consistent with recent new proof proposing that Kcnq3 channels might accelerate activity-dependent continuous changes in neuronal intrinsic excitability and structural plasticity that are well considered to form memory and addiction [46]. Our research demonstrated a functional characteristic for Kcnq3 channel-mediated inhibition in behaviorally relevant LH neurons significant for MA-induced drug addiction.

## 4. Materials and Methods

### 4.1. Animals

Conditional place preference experiments were conducted using C57BL/6 mice (20–25 g, 6–8 weeks old, male), which were purchased from the Laboratory Animal Center of Shandong University. Mice were reared in a 12 h light/12 h dark cycle, and all operations were in compliance with the regulations of the Animal Care and Use Committee of Shandong University.

### 4.2. Model Establishment and Drugs

We used black and white acrylic plates to make black and white boxes for the experiment. The apparatus contained two chambers, and the chambers were equal sized. The bottom of the box had a different floor texture, and the walls were either white or black. A white–black plate was used to isolate the white and black boxes with a gate to allow the mice to pass through. Mice were confined in a white box after MA (2 mg/kg, i.p.) [47] and in a black box after injection of normal saline. The training time was 30 min. On day 0, the baseline test (pre-test) was conducted for 15 min for every mouse. On days 2, 4, and 6, MA was injected into the mice, and then the mice were limited to the white chamber for 30 min. On days 1, 3, and 5, an equivalent volume of saline was injected into the mice, and then the mice were limited to the black chamber for 30 min. On day 7, the mice were allowed to explore the whole apparatus freely for 15 min (test). The time spent in the two boxes was recorded both pre-test and test [48], and CPP score was calculated as the difference between the time spent in the saline-paired compartment and the time spent in the MA-paired compartment (the time in the saline-paired compartment minus the time in the MA-paired compartment). For the inactivation of hM4Di, CNO (1 mg/kg, i.p.) was injected into the mice 30 min before the test.

### 4.3. Surgery

Chloral hydrate (400 mg/kg) was used to anesthetize the mice when the stereotaxic surgery was conducted. For intracellular calcium detection experiments, AAV2/9-CMV-GCaMP6s-P2A-nls-dTomato virus 300 nL (OBiO Technology Shanghai Corp., Ltd., Shanghai, China) was injected into the LH (anteroposterior (AP), −1.23 mm; mediolateral (ML), ±1.25 mm; dorsoventral (DV), 5.25 mm). For experiments that used chemogenetics to inhibit neurons in LH brain regions, AAV-hSyn-hM4Di-mCherry virus or the same viral vectors carrying mCherry alone (Shanghai Genechem Co., LTD., Shanghai, China) were injected into the bilateral LH brain region, and the injection volume was 400 nL. For the in vivo dendrite labeling experiment, mice were injected with a viral admixture bilaterally into the LH at a volume of 100 nL, which included a single pAAV copackage of DNA cassettes of CMV. Cre- and Cre-dependent EF1a DIO GFP (BrainCase, Shenzhen, China) was at a ratio of 1:1,000,000, respectively, so that the final viral admixture contained one virus with both cassettes for every 1,000,000 viruses that contained only the EF1a DIO GFP cassette. For the Kcnq3 overexpression experiment, mice were injected with pAAV-CMV-Kcnq3-WPRE or pAAV-CMV-Ctr without Kcnq3 bilaterally into the LH, and the injection volume was 400 nL. After surgery, mice were permitted to recover and express virus for at least 4 weeks before undergoing behavioral training.

### 4.4. Immunohistochemistry and Imaging

Mice were deeply anesthetized with chloral hydrate (400 mg/kg, i.p.) and then perfused with 0.9% saline solution and 4% PFA from the apex of the heart. Their brains were removed, sliced into 30 μm coronal sections using a vibratome (Leica Biosystems, Wetzlar, Deutschland, Germany), and collected in a six-well plate with PBS. For immunofluorescence staining, slices that included the LH region were blocked at room temperature with 0.3% Triton X-100 (PBS) and 10% normal goat serum for 30 min. Then, the cells were incubated with primary rabbit anti-c-Fos (1:1000 dilution; Abcam, Waltham, MA, USA), rabbit anti-Arc antibody (1:1000 dilution; Santa Cruz Biotechnology, Dallas, Texas, USA), rabbit anti-p-CREB (1:1000 dilution; Abcam, Waltham, MA, USA), and rabbit anti-Kcnq3 antibody (1:1000 dilution; Alomone Labs, Jerusalem, Israel) for 24 h at 4 °C. The sections were then washed three times in PBS and incubated with indicated secondary antibodies for 1.5 h at room temperature. The following secondary antibodies were used in our experiments: Alexa Fluor 594 goat anti-rabbit IgG (for c-Fos, p-CREB, Arc and Kcnq3, 1:200; ABclonal Technology, Wuhan, China), Alexa Fluor FITC goat anti-rabbit IgG (for c-Fos, 1:200; ABclonal Technology, Wuhan China). Finally, after three more washes with PBS, the sections were mounted on a microslide and imaged by a confocal microscope (Olympus, Tokyo, Japan). Three to six pictures were taken from different slices of 5 mice in each group. Quantification of labeled neurons was conducted with ZEN blue software with the same threshold.

### 4.5. Quantitative Real-Time PCR

Taking the LH region from the brains, 400 μL of R2 solution was added to each sample according to the instructions (Fastagen, Hefei, China), and the samples were immediately placed on ice for 1 min. The liquid in the tube was completely transferred to the corresponding inner tube of the double casing. Each tube was placed in a high-speed low-temperature centrifuge at 4 °C and 13,000 rpm for 1.5 min to discard the liquid in the outer casing. An equal amount of washing into the inner tube buffer was added, and centrifuged as above, which was repeated twice. Elute buffer (25–30 μL) was added to the inner casing to cover the entire bottom. The RNA samples were obtained by centrifugation. RNA purity was measured by a NanoDrop spectrophotometer. Reverse transcription of total RNA was performed using a PrimerScript RT reagent kit with gDNA Eraser (TAKARA, Beijing, China) according to the manufacturer’s instructions. qRT–PCR was conducted under the following conditions: 95 °C for 10 min, 95 °C for 10 s, and 60 °C for 30 s, totally repeated for 40 cycles with FastSYBR Mixture (CWbio, China). All results were normalized to GAPDH expression. The primer sequences were as follows: Kcnq3 (forward, 5′-GAGCCGACAAAGACGGGAC-3′; reverse, 5′-TTGGCGTTGTTCCTCTTGACT-3′).

### 4.6. Electrophysiological Recording

The brain tissue was removed rapidly and sliced into 300 μm coronal slices, including the LH region, using a vibratome (Leica) in cutting artificial cerebrospinal fluid (in mM: 112 NaCl, 3.1 KCl, 1.8 CaCl_2_, 0.4 KH_2_PO_4_, 12 MgCl_2_, 20 glucose, and 26 NaHCO_3_). Slices were incubated in oxygenated 34 °C artificial cerebrospinal fluid (in mM: 122 NaCl, 3.1 KCl, 1.8 CaCl_2_, 0.4 KH_2_PO_4_, 1.2 MgCl_2_, 20 glucose, and 26 NaHCO_3_) for at least 1 h before the experiment. Then, we conducted whole-cell patch-clamp recordings in voltage-clamp or current-clamp mode to record different electrophysiological indexes on neurons within the LH region. The resistance of the glass electrode used was 3–5 MΩ, which was filled with a liquid (pH 7.3), and the ingredients were (in mM): 135 potassium D-gluconate, 10 KCl, 10 HEPES, 0.1 EGTA, 0.5 Na-GTP, 2 Mg-ATP. Spontaneous currents were recorded at a holding potential of −70 mV, and evoked potentials were recorded at injections of 50 and 75 pA current stimulation in the perfusion liquid. Excitatory post-synaptic currents (EPSCs) were detected at a holding potential of −70 mV with 50 μM picrotoxin present in the artificial cerebrospinal fluid (ACSF) used for perfusion. During recordings, slices were continuously perfused with ACSF at a rate of ~2 mL/min and at 32 ± 1 °C. The data were analyzed using the Mini Analysis Program. All recording data were filtered at 2 kHz and digitized at 10 kHz. Data were acquired using digidata 1440 A and pCLAMP 10.6 software.

### 4.7. Golgi Staining

Golgi staining was conducted according to previous procedures [49]. Briefly, after the test, mice were perfused with 0.9% saline solution and PFA from the apex of the heart, and their brains were extracted and sliced into 5 mm sections. Tissues were impregnated by immersion in a standard Golgi-Cox solution containing 1% potassium dichromate, 1% mercuric chloride, and 0.8% potassium chromate for 7 d. Then, tissues were transferred to a 30% sucrose solution for 2 d and sectioned coronally into 150 µm sections, which were mounted on gelatinized slides and stained according to the Gibb and Kolb method. The slices were then covered with mounting media (Fisher Scientific, Pittsburgh, PA, USA) and deposited in the dark until the slices were completely dry. The appropriate LH brain slices were finally viewed under a microscope (Olympus-DP-72, Olympus, Tokyo, Japan). Neuron Studio software and Sholl analysis of ImageJ software were used to analyze neuronal spine density.

### 4.8. Quantification and Statistical Analysis

Experimental data are presented as the mean ± SEM, and statistical analysis was performed using GraphPad Prism 8.0 software. Comparisons of two groups of results were analyzed using the t test, while results from more than two groups were analyzed using one-way ANOVA. *p* < 0.05 was defined as a statistically significant difference.

## 5. Conclusions

In conclusion, our data demonstrated that the LH could regulate MA-mediated addictive behavior by acting on mechanisms involving synaptic plasticity and Kcnq3 channels. These results add a novel perspective on the MA-mediated addiction mechanism and suggest that LH neurons might be an efficacious target for the treatment of MA-induced spatial learning and drug-seeking in psychiatric disorders associated with MA addiction.

## Data Availability

The data presented in this study are available on request from the corresponding author.
